# Mobility-Aware Offloading Decision for Multi-Access Edge Computing in 5G Networks

**DOI:** 10.3390/s22072692

**Published:** 2022-03-31

**Authors:** Saeid Jahandar, Lida Kouhalvandi, Ibraheem Shayea, Mustafa Ergen, Marwan Hadri Azmi, Hafizal Mohamad

**Affiliations:** 1 Electronics and Communication Engineering Department, Faculty of Electrical and Electronics Engineering, Istanbul Technical University (ITU), Istanbul 34467, Turkey; bonab18@itu.edu.tr (S.J.); mustafaergen@gmail.com (M.E.); 2 Department of Electrical and Electronics Engineering, Dogus University, Istanbul 34775, Turkey; lida.kouhalvandi@ieee.org; 3 School of Electrical Engineering, Faculty of Engineering, Universiti Teknologi Malaysia, Johor Bahru 81310, Malaysia; hadri@utm.my; 4 Faculty of Engineering and Built Environment, Universiti Sains Islam Malaysia, Nilai 71800, Malaysia; hafizal@usim.edu.my

**Keywords:** fifth generation (5G), sixth generation (6G), handover (HO), multi-access edge computing (MEC), mobility management, task offloading (TO)

## Abstract

Multi-access edge computing (MEC) is a key technology in the fifth generation (5G) of mobile networks. MEC optimizes communication and computation resources by hosting the application process close to the user equipment (UE) in network edges. The key characteristics of MEC are its ultra-low latency response and real-time applications in emerging 5G networks. However, one of the main challenges in MEC-enabled 5G networks is that MEC servers are distributed within the ultra-dense network. Hence, it is an issue to manage user mobility within ultra-dense MEC coverage, which causes frequent handover. In this study, our purposed algorithms include the handover cost while having optimum offloading decisions. The contribution of this research is to choose optimum parameters in optimization function while considering handover, delay, and energy costs. In this study, it assumed that the upcoming future tasks are unknown and online task offloading (TO) decisions are considered. Generally, two scenarios are considered. In the first one, called the online UE-BS algorithm, the users have both user-side and base station-side (BS) information. Because the BS information is available, it is possible to calculate the optimum BS for offloading and there would be no handover. However, in the second one, called the BS-learning algorithm, the users only have user-side information. This means the users need to learn time and energy costs throughout the observation and select optimum BS based on it. In the results section, we compare our proposed algorithm with recently published literature. Additionally, to evaluate the performance it is compared with the optimum offline solution and two baseline scenarios. The simulation results indicate that the proposed methods outperform the overall system performance.

## 1. Introduction

The technological evolution is currently increasing with the creation of new and up-to-date technology that obtains information from carried data. Based on Cisco’s new annual Internet report, 5G will support more than 10% of the world’s mobile connections by 2023. There will be nearly 30 billion devices/connections by 2023—5% of those will be mobile [1].

The emergence of the modern multi-access edge computing (MEC) technology is a key discovery in the fifth generation (5G) and future sixth generation (6G)mobile networks because it optimises communication and computation resources effectively [2,3,4,5,6]. This process employs edge resources near the user equipment (UE) to reduce latency while enhancing reliability and stability [7,8]. The offloading task sends computational tasks/data to the MEC server for processing [9,10]. Once the MEC executes the received task, it is responsible for sending the results back to the appropriate user. Edge computing has several advantages, such as decreased end-to-end latency, increased multimedia bandwidth, computation-rich resources, and enhanced flexibility. The drawback with most UEs is resource limitation due to computation requirements, even though new and improved hardware is available. However, MEC could solve this issue by hosting the computational tasks.

This study proposes two algorithms for choosing the best parameters in the optimisation function. Online algorithms are used to enhance the performance evaluation. The handover (HO) time cost is also considered in the formulation of the optimisation function while making optimum offloading decisions. The two algorithms presented in this work assume that the upcoming future tasks are unknown, and online task offloading (TO) decisions are therefore considered. The proposed methods are not model-based and can be implemented in any mobility scenario. In the first algorithm, called the online UE-base station (BS) algorithm, the users have both the user-side and BS-side information. In the second algorithm, BS-learning, users only have user-side information. The overall simulations are conducted using the MATLAB tool, and are verified in the case where the BS-side information is known. Our proposed method exhibits near to optimum performance. For the scenario based on observation and learning, the results reveal a decrease in performance due to additional costs, such as the HO time cost. The energy performance is also compared with other related papers, proving that in terms of the energy budget, our two proposed methods exhibit near to the optimum solution.

The remainder of this paper is organised as follows: A comprehensive literature review with the motivation of this work is provided in Section 2. Section 3 provides the system model analysis. Section 4 presents the problem formulation and the optimisation problem. Section 5 discusses the proposed online task offloading decision algorithms, problem formulation, and proposed online algorithms. Section 6 displays the simulation setting and numerical results. Finally, Section 7 includes the conclusion of this paper and additionally future tasks that can be employed for improving this manuscript.

## 2. Literature Review and Motivation of This Work

### 2.1. Literature Review

Recently, various studies have been published in the MEC domain, and this section provides a comprehensive review of these publications.

The mobility management that has been widely used in various applications such as IoT generates realistic mobility patterns [11,12]. In [13], a mobility-aware hybrid flow rule cache scheme is presented for tackling the problem of forwarding node. From another point of view, edge computing can provide the solutions for the cloud limitations in the current communication systems [14].

**Figure 1:**
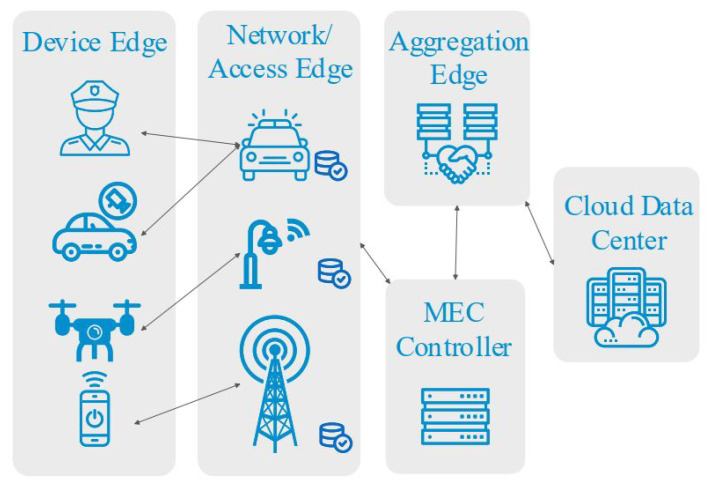
From cloud to network edges.

Latency factor is an important specification in a vehicular network that results in delays, generated from the high mobility of vehicles [15,16]. Data traffic is expanding with the increase in massive connectivity. The favourable characteristics of MEC technology are suitable for emerging 5G networks as they include ultra-low latency response and real-time applications [17,18]. It is expected that mobile broadband will increase to 8 billion by 2025. In 2017, the European Telecommunications Standards Institute (ETSI) changed the name ‘mobile edge computing’ to ‘multi-access edge computing’ for addressing non-cellular operators. MEC servers can now be deployed with a radio access network (RAN), BSs, Wi-Fi access points, and fixed connections [19]. The 3GPP includes MEC technology in the 5G network with the technical specification of 3GPP TS 23.051. Figure 1 presents a comprehensive summary of MEC servers with RAN, BSs (e.g., 4G, 5G, and 6G), Wi-Fi access points, and fixed connections.

The MEC technology can be employed in various applications such as video analytics, location services, and data caching, which allows for flexible and rapid innovative applications. It can also be used in an autonomous vehicular network, sharing information with roadside units (RSU) and pedestrians without involving any cloud servers [20,21,22]. The development of MEC at the network edge is critical. The devices and sensors of the IoT require significant storage, computation resources, and suitable bandwidth, as the ever-increasing data have caused limited computational resources [23].

The MEC technology has two main challenges [4,9]. The first challenge is appropriately allocating MEC resources, as multiple users are within the coverage region of the MEC server. Once the computational resource is allocated to a user, it will then decide whether to offload the task or execute it locally. Multiple BSs are available for a single user. Choosing the optimum BS for offloading is the current research topic in various MEC-related studies [24,25,26,27,28]. The most common solution is to develop a utility cost function that determines the optimum solution based on the optimisation methods. Because resource allocation, communication, computation, and user mobility must all be considered, joint optimisation problems and analysis should be accomplished. Most joint problems are non-convex and NP-hard [29,30,31].

The second main challenge in MEC-enabled 5G networks is that MEC servers are distributed within ultra-dense networks [3,32]. Managing user mobility within the small-scale coverage of the MEC server is a significant issue [33,34,35,36]. In the recently published literature, MEC often neglects user mobility and assumes they are constant due to complexity [37,38,39]. Table 1 and Table 2 present the summary of mobility management and the related studies in this field in a comprehensive view, respectively. Table 2 demonstrates that our proposed method, in comparison with other reported methods including machine learning approach, is advanced as energy consumption of UEs, handover delay, and task offloading are concurrently considered. In simple words, what is lacking in the previous studies is concurrently considering these parameters. Our method leads the way of designers in mobility-aware offloading decisions.

**Table 1:** Summary of mobility management challenges in MEC.

Ref.	Topic	Challenges
[40]	Pre-configuration	- Pre-allocation: To enable MEC systems for reducing end-to-end delay during high mobility; - Reallocation group: To share application information among MEC server for HO scenario.
[4,41]	HO prediction	- To choose essentially an optimum server sequentially as vehicles move; - To study the HO in urban areas due to the higher uncertainty.
[42,43]	Densification	- To manage the mobility of users in an ultra dense network (UDN); - To provide small-scale coverage results: frequent HOs, HO process power consumption, and radio resource constraint; - To prepare real-time information for long-term optimisation.
[44]	Network diversity	- To offload tasks to MEC servers over 2G, 3G, 4G, 5G, WLAN, or and overlapping mobile WiMAX networks by UEs. (These various types of networks causes overlapping).
[44,45]	Self optimization	- To make HO decision in MEC, where the HO control parameter needs to be assigned optimally; - To optimise both HO and task offloading in MEC result conflicts in optimisation function.

**Table 2:** Summary of related studies on handover decision optimisation in MEC.

Ref.	Topic	HO	TO	Limits	Contribution
[41]	Task offloading	Yes	Yes	The energy consumption of UEs are not considered.	- Task offloading decision was formulated as MDP to minimize delay considering handover, migration, communication and computation; - Addressed the uncertainty of transition probabilities.
[4]	Joint optimisation	Yes	Yes	- Only one handover is considered for each UE; - Not applicable for 3D random mobility (limited for 1D road)	- Mobility-aware computation offloading was proposed in MEC-based vehicular networks.
[24,46]	Joint optimisation	No	Yes	- No handover; - No mobility model.	Service caching placement and computation offloading is considered.
[25,29]	Proactive network association	No	Yes	- No handover; - No mobility model; - No UE energy constraint.	- Online proactive caching is considered; - Based on the MDP and Lyapunov optimisation, a two-stage online decision algorithm for proactive network association was innovated.
[9]	Task offloading	No	Yes	Mobility model has no handover cost (i.e., dedicated as a future work)	- Mobility-aware multi-user offloading optimisation for MEC; - UEs energy consumption is included.
[30]	Handover management	Yes	Yes	The system model only reduces number of handovers No handover delay or cost is optimized.	Existing work focused on cloudlet placement and user-to-cloudlet association problem.
[43]	Ultra-dense network	Yes	Yes	The parameter in optimization function leads into sub-optimum MEC and lower performance	Minimized average delay subjected to communication, computation, and handover under the limited energy budget of users.
[31]	Ultra-dense network	No	Yes	No handover (mentioned as challenge but not considered).	Computation offloading for multi-access MEC in UDN is investigated
[8]	Fog computing	No	Yes	Mobility model has no handover cost (mentioned as challenge but not considered)	Task offloading and migration schemes are studied in fog computing
[33]	Fog computing	Yes	Yes	The energy consumption of UEs is not considered (i.e., dedicated as a future work)	- Offloading delay and handover cost are considered as performance metrics; - The RSU considered the targeted node based on vehicle mobility and dynamic computation resources.
[47]	Deep reinforcement learning task scheduling	No	Yes	The tasks are completed before handover (i.e., only controls HO) No handover cost	- DRL is used for offloading scheduling in VEC; - The trade-off between task latency and energy consumption is considered by scheduling tasks to wait in the queue.
[48]	Mobility management using reinforcement learning	Yes	Yes	The energy consumption of UEs is not considered.	An online RL was proposed to optimize handover decisions by predicting user movement trajectory and periodic characteristics of the number of users.
[49]	Edge autonomous energy management	No	No	Only energy managed	An RL-based droplet framework is used Droplets learn energy consumption statistics of the devices.
Our work	Mobility-aware offloading decision	Yes	Yes	In the offloading process, the BSs are considered to be all ON.	- Concurrently considering HO delay, UE energy, and TO. - Optimized parameters are used to increase overall performance. - User-centric approach learns BS-side information without prior knowledge.

The conventional MEC-based offloading methods are not powerful enough, and advanced optimisation methods are required. Xuefei presents a method, the Markov decision process (MDP), for reducing the amount of delay in high mobility vehicular networks [41]. Another method of joint optimisation is presented in [50,51] for vehicle random mobility. In [52], the boundless simulation area (BSA) model for configuring the vehicles’s mobility is presented, which is based on the Markov chain. For the IoT technology, a proactive network association can be a sufficient solution for reducing latency in MEC systems [53,54]. As a solution for reducing the handover in MEC-based networks, in [55], a region partitioning approach is presented, where this method is evaluated for both random and real traces. In another work [56], the dynamic service placement is employed, where offline algorithms are used for determining the optimal service for minimizing the average cost. To support the energy-constrained devices such as IoT sensors, the wireless powered MEC networks are integrated with simultaneous wireless information and power transmission (SWIPT) techniques in [57]. In another work [58], the task offloading and wireless power transformation (WPT) are jointly optimized. Moreover, the authors proposed a resource allocation scheme to minimize energy consumption.

There are important limitations in the previous summarized literature in that these works do not include HO cost in the optimisation function. Additionally, either energy factor or delay is considered in these methods where the optimum parameter cannot be selected effectively.

### 2.2. Motivation of This Work

This work considers user mobility while having optimum offloading decisions. To support service continuity, the host MEC should reallocate the task to the target MEC server. During the task offloading, if users move from the coverage region of a base station, it will cause handover that leads to additional cost. Hence, our problem formulation includes the handover cost. Below is a summary of the two employed algorithms.

In the first algorithm, calculating the optimum BS for offloading is possible because the BS information is available. No handover will be presented, making the performance close to the optimal offline solution. However, we must ensure that users’ total energy consumption will not exceed its budget as the energy budget is limited.

In the second algorithm, the BS-side information is not available. This means that the channel gains and the available computing CPUs are unknown. Therefore, users must learn time and energy costs through observations. The optimum BS is determined based on these observations. However, sub-optimum BS selection may occur due to the variance of observation. Selecting sub-optimal BS will increase the total cost and number of handovers. Therefore, it is essential to select optimum controlling parameters in the optimisation function.

The novelty and contribution of this manuscript can be summarized as follows:

- Our proposed method is beneficial for the designers in the field of mobility management in MEC, as this approach considers three important parameters—energy consumption of UEs, handover delay, and task offloading—concurrently. This jointly consideration is missing in the reported literature;
- The proposed method is intelligent enough for finding the optimal value of alpha presented in Equation (18), resulting in average time and total energy costs near to the optimum offline solution;
- In our proposed methods, the offloading decision is user-centric, which is decided on the UE-side. Moreover, Algorithm 2 initially has neither base station nor network information. In this case, the learning process is used with optimized steps to reduce overall delay and improve energy performance.

## 3. System Model

**Figure 2:**
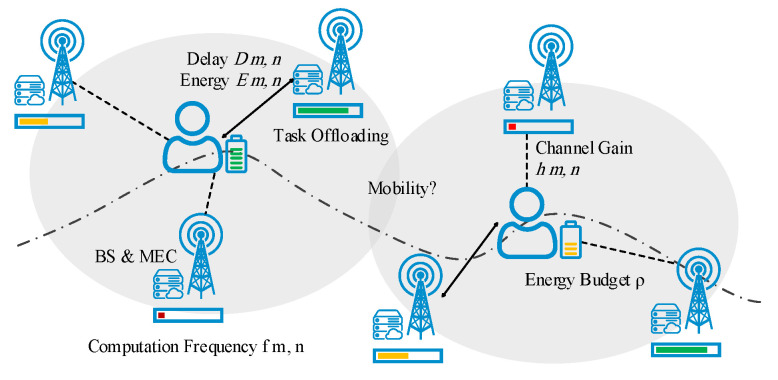
System model of mobility-aware task offloading in MEC enabled 5G network.

The MEC network consists of the UEs and *N* BSs equipped with the MEC server. The BSs are distributed on a finite two dimensional (2D) regular grid network, each with a supporting radius of R (as shown in Figure 2). Let N={1,2,…,N} denote the index set of MEC servers, and $l_{n}$ represents the location of BS *n* equipped with the MEC server. The mobile UEs create a total *M* task for offloading into MEC servers, denoted by the set of M={1,2,…,M}. Let $l_{m}$ signify the location of task *m* created by a user with mobility. Due to the densified deployment of BSs, it is assumed that each task *m* can be served by multiple BSs. The set of admissible BSs to location $l_{m}$ can be represented as

(1) $$A\left(m\right)=\{n|\|l_{n}-l_{m}\|\leq R,\forall n\in N\}$$

The users decide which candidate BSs A(m)∈N are appropriate to offload computational tasks based on the utility cost. It is also assumed that only one BS is responsible for computing each task *m*. Hence, users will choose one BS station from A(m) for each task *m*. Due to large task sizes, each task *m* can be divided into many sub-tasks. An online learning approach is considered to manage the user mobility. Any random mobility model can be applicable and suitable. The system model has been divided into three sub-models: computation task model, network model, and mobility model. The explanations of each sub-model follow.

### 3.1. Computation Task Model

Here, it is supposed that a UE generates task *m* to offload into the MEC server. The computational tasks can be parameterized as triplets in (2):

(2) $$\chi ≜[d_{m},c_{m},t_{m}]$$

where $d_{m}$ specifies the total data size of task *m* (in bits). The computation intensity $c_{m}$ refers to the number of required CPUs to accomplish computing one-bit data of a task. The $t_{m}$ indicates the computation deadline time allowed for executing task *m* (in seconds). As the size of the computed result is generally small, it is omitted from the equation. To consider large input data sizes, $d_{m}$ can be divided into $k_{m}$ number of equal-sized sub-tasks. Therefore, $d_{m}=k_{m}d_{sub}$, and $d_{sub}$ denote to each sub-task data size.

As the UEs decide to offload the computational task, the MEC server is responsible for resource allocation based on available resource constraints. The computation resource inside the MEC server is indicated based on the CPU cycle frequency. Let F(n) represent the total computing constraint of the MEC server *n*, which indicates the maximum available CPU cycle frequency of the MEC server. The computation frequency $f_{m,n}$ (denoted as CPU frequency) is the total resource allocated by MEC server *n* into the UE with requesting task *m* for computation. Accordingly, the time cost for executing a sub-task of *m* by the MEC server *n* is given as (3):

(3) $$t_{m,n}^{e}=d_{sub}\times \frac{c_{m}}{f_{m,n}}$$

where $d_{sub}$ specifies the sub-task data size. Once all sub-tasks are computed by MEC server *m*, which takes a total duration of $T_{m,n}^{tot}=\sum_{k=1}^{k_{m}}t_{m,n}^{e}$, it deallocates resources so other UEs could use deallocated CPU for their tasks.

### 3.2. Network Model

In this study, it is assumed that channel bandwidth ω is equally allocated among tasks. According to [59], the data transmission rate from the UE with task *m* to the BS *n* is obtained by (4):

(4) $$r_{m,n}=\omega \times \log_{2}\left(1+\frac{p_{m}h_{m,n}}{N_{\sigma^{2}}}\right)$$

where $N_{\sigma^{2}}$ represents the noise power, $p_{m}$ is the transmission power of UE *m*, and $h_{m,n}$ denotes the wireless channel gain from UE with task *m* to BS *n* at location $l_{m}$, respectively. It is assumed that users are constant during task offloading. As each task *m* can be divided into multiples sub-tasks, the channel gain $h_{m,n}$ will remain constant. The wireless channel gain $h_{m,n}$ is described as (5):

(5) $$h_{m,n}\left[dB\right]=127+30\times \log d\left[Km\right]$$

where $d=\|l_{n}-l_{m}\|$ refers to the distance between task *m* at location $l_{m}$ and BS *n* (in kilometers). The channel model is as suggested in [60], which is for ultra-dense heterogeneous networks, and the fading follows a Rayleigh distribution.

Additionally, the time and energy costs of offloading a sub-task of *m* to BS *n*, respectively, can be presented using (6) and (7):

(6) $$t_{m,n}^{o}=\frac{d_{sub}}{r_{m,n}}$$

(7) $$e_{m,n}^{o}=p_{m}t_{m,n}^{o}$$

where $p_{m}$ specifies power required to transmit *m*. The $p_{m}$ is assumed as given for each task, and it depends on many parameters, including antenna gain.

### 3.3. Mobility Model

The large data size of tasks can be divided into multiple sub-tasks. Each sub-task can be executed in various BSs. Changing the BSs while computing sub-tasks of *m* can result in HO delay cost.

Let $\tau_{m}^{h}$ denote HO delay of a one-time BS switch. Considering the multiple sub-tasks, the sequence of BS is represented by $\eta_{m}=\left\{\eta^{1},\eta^{2},\ldots ,\eta^{k_{m}}\right\}$. According to [43], the HO delay for all sub-tasks of *m* can be calculated as follows:

(8) $$t_{m,n}^{h}=\tau_{m}^{h}\sum_{k=2}^{k_{m}}H\left\{x\right\}$$

(9) $$H\left\{x\right\}=\left\{\begin{array}{cc} 1 & \eta^{k}\neq \eta^{k-1},\;\;\;\;\eta^{k}\in A\left(m\right)\\ 0 & \mathrm{otherwise} \end{array}\right.$$

## 4. Problem Formulation

In the mobile system, the UE experience during offloading decisions is determined by both latency and energy budget. A utility function must be designed as a trade-off between the time and energy budgets to make the appropriate decision when the BS is to offload and perform HO due to the UE mobility. The total time cost for task *m* can be calculated as the sum of execution time, task offloading time, and HO time, as in (10):

(10) $$T_{m,\eta }^{tot}=\sum_{k=1}^{k_{m}}\left(t_{m,\eta }^{e}+t_{m,\eta }^{o}\right)+t_{m,\eta }^{h}.$$

The total time cost can be written as (11):

(11) $$T_{m,\eta }^{tot}=\sum_{k=1}^{k_{m}}\left(t_{m,\eta }^{e}+t_{m,\eta }^{o}+\tau_{m}^{h}H\left\{x\right\}\right).$$

This study only considers the task offloading energy cost of UEs. The total energy consumption of UE for offloading task m is calculated by

(12) $$E_{m,\eta }^{tot}=\sum_{k=1}^{k_{m}}e_{m,\eta }^{o}.$$

The decision-making process cannot predict the UE trajectory after the computation deadline time $t_{m}$ while considering UE mobility during task offloading. Therefore, the total time cost needs to be less than the time constraint $t_{m}$ presented as

(13) $$T_{m,\eta }^{tot}\leq t_{m},\forall m\in M$$

Eventually, the UE’s limited energy budget will restrict the sum of the energy cost *M* tasks to be less than energy constraint ρ. The total UE energy is constrained as below:

(14) $$\sum_{m=1}^{M}E_{m,\eta }^{tot}\leq \rho .$$

As previously noted in Equation (1), all candidate BS η for task *m* should be within coverage area with a radius of *R*, denoted as (15):

(15) $$\eta_{m}^{k}\in A\left(m\right),\forall k\in \left\{1,2,\ldots ,k_{m}\right\}.$$

Our target is to determine the optimal offloading decision to minimize the total time cost within a limited energy budget for all tasks *M*. The problem is formulated as follows (16):

(16) $$\mathrm{GP}:\min_{\eta }\frac{1}{M}\sum_{m=1}^{M}T_{m,\eta }^{tot}$$

where $\eta \in \{\eta_{1},\eta_{2},\ldots ,\eta_{M}\}$ is the variable vectors to be optimised. Constraint (13) ensures that the task will be executed on time (subject to the computation deadline time $t_{m}$). Constraint (14) guarantees that the energy consumption is below the energy budget ρ. Additionally, constraint (15) chooses all the candidate BSs that can serve task *m*.

Due to the non-linearity of the optimisation problem presented in (16) and the complexity of other variables, the GP is a mixed-integer non-linear programming (MINLP) problem [61]. An online algorithm is proposed to address the UEs’ trajectory during task offloading.

## 5. Online Task Offloading Decision Algorithm

This section presents two mobility-aware online task offloading decisions for MEC, based on the Lyapunov optimisation and without the knowledge of future tasks. The proposed online algorithms are then compared with the offline optimal solution.

### 5.1. Mobility-Aware UE-BS Algorithm

This algorithm simultaneously has UEs with both the UE-side state information and the BS-side information. The BS remains constant during the offloading of one task, as previously noted. Having both sides’ state information helps the UE to select the optimal BS for offloading and avoids any HOs. Within the UE-BS scenario, all sub-tasks of *m* will be served by a single optimum BS $\eta_{m}^{opt}$. The main challenge for solving GP in an online algorithm is that without having future task information, m+1,m+2,…,M, the limited energy budget for current tasks will be spent and nothing will remain for upcoming tasks. To overcome this issue, the solution is to define an energy queue and store the used energy budget. By exceeding a specific amount of energy budget for task *m*, future tasks will be allocated to another candidate BS η∈A. According to [43], the energy queue is obtained as (17):

(17) $$\beta_{m+1}=\max\left\{\beta_{m}+E_{m,\eta_{m}^{opt}}^{tot}-\rho /M,0\right\}$$

where $\beta_{0}$ is equal to zero. By using the Lyapunov optimisation, we can define the optimisation problem as a trade-off between time cost and energy cost of task offloading, represented as (18):

(18) $$Z_{m}^{tot}=\alpha T_{m,n}^{tot}+\beta_{m}E_{m,n}^{tot}$$

where α is a control parameter to adjust the trade-off between energy and time cost. No handover will occur as we have both sides’ information in the UE-BS scenario and we selected the appropriate BS for the entire time of processing task *m*. The total time cost can be reduced to $T_{m,\eta }^{tot}=\sum_{k=1}^{k_{m}}\left(t_{m,\eta }^{e}+t_{m,\eta }^{o}\right)$. As all sub-tasks will be served by a single optimum BS $\eta_{m}^{opt}$, the optimisation function in (18), can be simplified as

(19) $$z_{m}=\alpha \left(t_{m,\eta_{m}^{opt}}^{e}+t_{m,\eta_{m}^{opt}}^{o}\right)+\beta_{m}e_{m,\eta_{m}^{opt}}^{o}.$$

To minimize the cost $z_{m}$ in Equation (19) for each task *m*, the online UE-BS algorithm presented in Algorithm 1 is proposed.
 **Algorithm 1:** Mobility-aware online UE-BS algorithm  
**Input:**
A(m), $d_{m}$, $c_{m}$, $f_{m,n}$, $h_{m,n}$ and α   
1: **if**
m=fJ+1,∀f=0,1,…,F−1 
**then**   
2:  $\beta_{m}\leftarrow 0$   
3: **end if**   
4: Choose $\eta_{m}^{*}$ subject to (13), (15) by solving
     $\min_{n\in A(m)}\alpha \left(t_{m,\eta_{m}^{opt}}^{e}+t_{m,\eta_{m}^{opt}}^{o}\right)+\beta_{m}e_{m,\eta_{m}^{opt}}^{o}$   
5: Update $\beta_{m}$ according to (17).

Because there is no handover within this scenario, there is no need to divide tasks into sub-tasks. Therefore, according to the optimization function in line 4, the Algorithm 1 complexity only relies on the number of candidate BS A(m), which is O(|A(m)|).

### 5.2. Mobility-Aware BS Learning Algorithm

In this algorithm, the UEs only have the UE-side state information. UEs are required to learn the BS state information to make offloading decisions. Unlike the UE-BS algorithm where all UEs stick to a single and optimum BS for all sub-tasks of *m*, the learning process causes sub-optimal BS selection. Choosing sub-optimal BS will cause additional cost as well as HOs during the learning process.

One solution for learning optimal BS is to offload all sub-tasks of *m* to every candidate BS $\eta_{m}\in A\left(m\right)$ and to observe the total energy and time costs. Let ${\tilde{t}}_{m,n}$ and ${\tilde{e}}_{m,n}$ represent the observed time cost and energy cost, respectively. Based on Equation (18), the observed optimisation function is

(20) $${\tilde{z}}_{m,n}=\alpha {\tilde{t}}_{m,n}+\beta_{m}{\tilde{e}}_{m,n}$$

where ${\tilde{z}}_{m,n}$ is a noisy version of $z_{m,n}$ with a specified variance. The main challenge in the BS learning scenario is that UEs may offload their sub-tasks into sub-optimal BSs due to the variance of $\tilde{z}$. The UE will try to offload their tasks into all BSs to learn the best optimum BS. However, offloading many tasks is not practical and may cause frequent HOs. A possible solution is to assign a stop parameter for the learning process. Therefore, the algorithm will only apply to the first $k_{s}$ sub-task, and the remaining sub-tasks will be offloaded to pre-determined optimal BS. The second challenge is choosing the stop learning parameter. The large $k_{s}$ may cause frequent HOs and may increase costs, whereas the small $k_{s}$ will lead to the selection of sub-optimal BS. The proposed Algorithm 2 presents the online UE-BS algorithm for minimising the cost ${\tilde{z}}_{m,n}$ in Equation (20) for each task *m*.
 **Algorithm 2:** Mobility-aware online BS learning algorithm  
**Input:**
A(m), $d_{m}$, $c_{m}$ and α.
   
1: **if**
m=fJ+1,∀f=0,1,...,F−1
**then**
   
2:  $\beta_{m}\leftarrow 0$
   
3: **end if**
   
4: **for**
k=1,…,|A(m)|
**do**
   
5:  connect o each BS n∈A(m) once.
   
6:  Update ${\bar{z}}_{m,n,k}=\alpha {\tilde{t}}_{m,n}+\beta_{m}{\tilde{e}}_{m,n}$.
   
7:  Update $\theta_{m,n,k}=1$.
   
8: **end for**
   
9: **for** 
$\left|A\left(m\right)\right|+1,...,k_{s}$
**do**
   
10:  Connect to $\eta_{m}^{k}=\arg\min_{n}\left\{{\bar{z}}_{m,\eta_{m}^{k},k}-\beta \sqrt{\frac{2\ln k}{\theta_{m,n,k}}}\right\}$
   
11:  Observe ${\tilde{t}}_{m,\eta_{m}^{k}}$ and ${\tilde{e}}_{m,\eta_{m}^{k}}$.
   
12:  ${\bar{z}}_{m,\eta_{m}^{k},k}\leftarrow \frac{\theta_{m,\eta_{m}^{k},k}{\bar{z}}_{m,\eta_{m}^{k},k}+\alpha {\tilde{t}}_{m,\eta_{m}^{k}}+\beta_{m}{\tilde{e}}_{m,\eta_{m}^{k}}}{\theta_{m,\eta_{m}^{k},k}+1}$.
   
13:  $\theta_{m,\eta_{m}^{k},k}\leftarrow \theta_{m,\eta_{m}^{k},k}+1$.
   
14: **end for**
   
15: **for**
$k_{s}+1,\ldots ,k_{m}$
**do**
   
16:  Connect to $\eta_{m}^{k_{s}}$, $\forall k\in \{k_{s}+1,k_{s}+2,\ldots ,k_{m}\}$
   
17: **end for**
   
18: Update $\beta_{m}$ according to (17).


In Algorithm 2, the task is divided into $k_{m}$ number of sub-tasks. Therefore Algorithm 2 has service continuity despite handover because of dividing the task into small-sized sub-tasks. However, these division introduces additional complexity, as seen in Algorithm 2. As noted above, the algorithm only applies into $k_{s}$ number of tasks, which results in computation complexity of $O(k_{s}|A\left(m\right)|)$. This means the small learning step $k_{s}$ leads to lower complexity. However, as noted above, the lower the $k_{s}$ means sub-optimal BSs may be selected. Therefore, there is a trade-off between having optimum BS and lower computation complexity in the algorithm.

## 6. Simulations Results and Discussion

**Figure 3:**
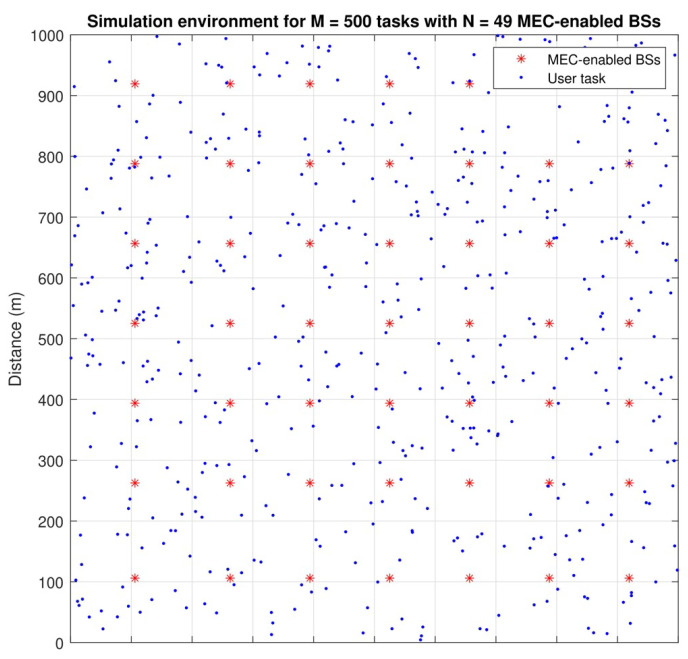
The location of BSs and users within simulation region.

This section provides the simulation experiments for evaluating the proposed methods. Table 3 summarises the main simulation parameters with their values. The coverage region of each MEC-equipped BS is considered to be a circle with a radius of 150 m deployed on a regular grid network within an area of 1000×1000 m^2^, as shown in Figure 3. The channel bandwidth of BS is set to be ω=20 MHz. The channel gain from the UE to BS is modelled as 127+30logd (km) dB [60]. All wireless communication parameters are set based on the 3GPP specification [62].

According to [43], the one-second video size should be set to $d_{sub}=0.62$ Mbits. Let us assume that each video is 60 s to 120 s long, and $k_{m}\in \left\{60,\ldots ,120\right\}$. According to the following definition, $d_{m}=k_{m}d_{sub}$, the input data size is uniformly distributed $d_{m}∼U\left(37.2,74.4\right)$ MBits. The same consideration holds for computation intensity, which is uniformly distributed within U(500, 1000) cycles/bits. Each MEC server *m* allocates its available computational CPU resource to its users ($f_{m,n}$), and the total used CPU should not reach the resource constraint of that MEC server ($F_{n}$). The MEC server *n* uniformly distributes the resource constraint $F_{n}=20$ GHz to its UEs. For simplicity, it is assumed that the available CPU frequency on all BSs are equal and are set to 25 GHz. To ensure that execution of each sub-task is completed within its latency constraint, $t_{m}$ is set to be 150 ms. After reaching the total execution time of a task $T_{m,n}^{tot}=\sum_{k=1}^{k_{m}}t_{m,n}^{e}$, the MEC server deallocates the resource $f_{m,n}$ and makes it available for reallocation to other users as soon as they request it.

The number of tasks varies for different simulations. We consider a fixed number of BSs as N=49, where all are active the entire time. It is also assumed that there is at least one BS to provide service to each UE. The transmit power of mobile UEs are $p_{m}=0.5$ W, and the noise power is set to be $N_{\sigma^{2}}=2\times 10^{-13}$ W. The one time HO is $\tau_{m}^{h}=5$ ms, and the battery capacity is J=1 kJ.

This section compares our two proposed methods, UE-BS and BS-learning algorithms, with the optimum offline solution as well as the two common benchmark algorithms known as time greedy and energy greedy. The performance of the proposed methods are evaluated and compared with the related study of [43].

**Figure 4:**
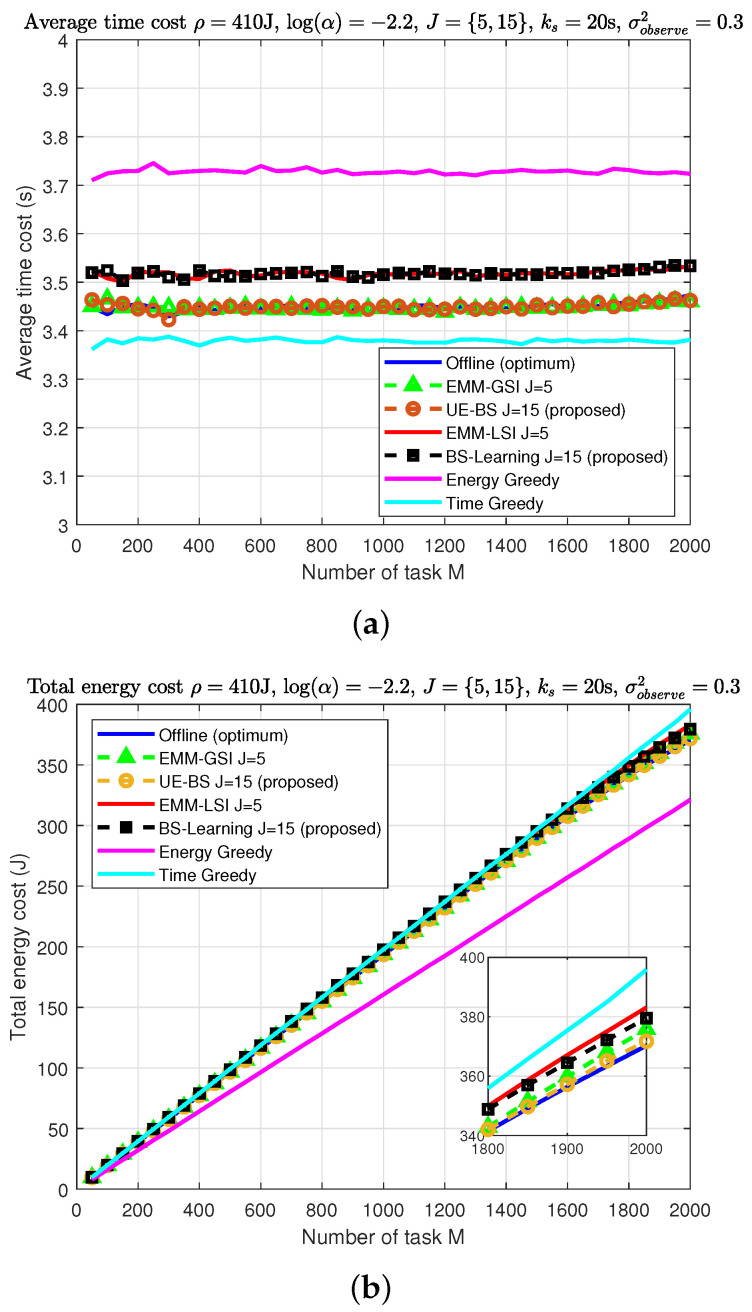
Performance evaluation of algorithms (ρ=410 J, log(α)=−2.2, J={5,15}, $k_{s}=20$ s, $\sigma_{observe}^{2}=0.3$): (**a**) average time cost; (**b**) total energy cost.

Figure 4 compares the average time cost and total energy cost for all algorithms over different task sizes M. As seen in Figure 4a, both EMM-GSI and the proposed UE-BS algorithms have their time cost close to the optimum offline solution due to possessing BS-side information. However, EMM-LSI and the proposed BS-learning have additional costs due to sub-optimal BS selection and handover cost.

For the higher number of task size in Figure 4b, the proposed methods exhibit slightly better performance compared to existing studies that have energy cost near optimum for the offline solution. The proposed methods maintain energy consumption below the energy budget ρ while achieving relatively low time cost.

**Figure 5:**
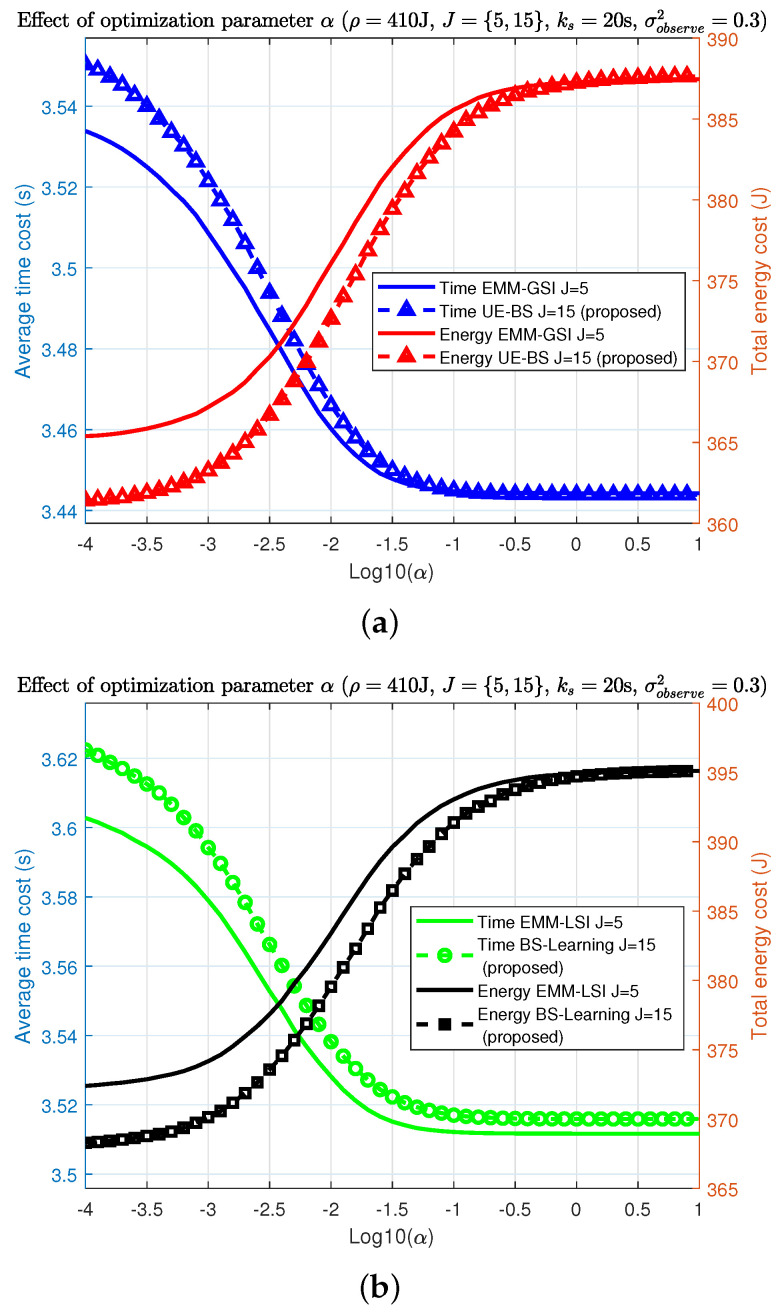
Effect of optimization parameter α (ρ=410 J, J={5,15}, $k_{s}=20$ s, $\sigma_{observe}^{2}=0.3$): (**a**) online UE-BS algorithm; (**b**) online BS-learning algorithm.

Figure 5 displays the trade-off between the average time cost and the total energy cost for α from $10^{-4}$ to 10. The α is the controlling parameter in the optimisation solution. We must therefore ensure it is optimally selected. The interception points indicate the optimum α values, as presented in Figure 5.

In Figure 5a, the BS-side information is presented. In Figure 5b, the proposed algorithm learns the total cost throughout observations. As can be seen, the interception points are almost the same, varying between [−2.3,−2.1]. To conveniently evaluate all algorithms in the same condition, the controlling parameter is set to be log(α)=−2.2. Due to the HO and learning process in Figure 5b, the time and energy costs are slightly higher than those in Figure 5a.

**Figure 6:**
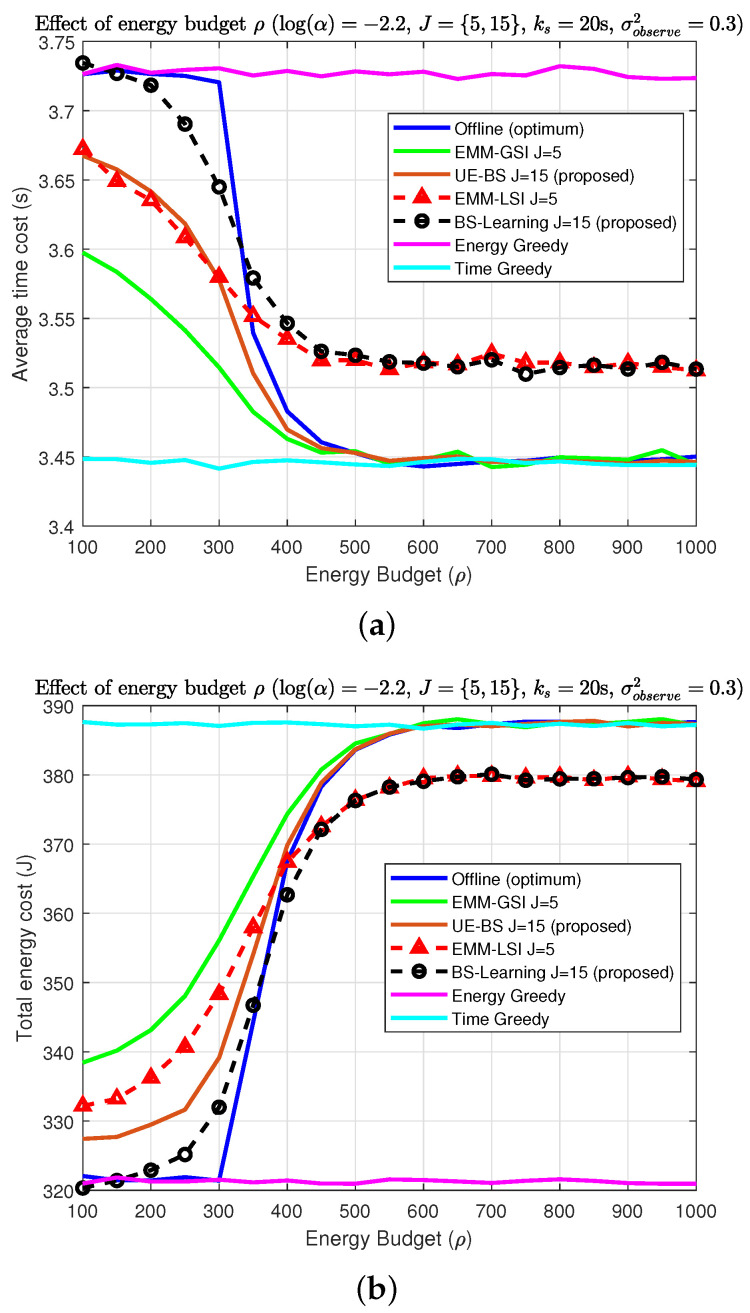
Effect of energy budget ρ (log(α)=−2.2, J={5,15}, $k_{s}=$ 20 s, $\sigma_{observe}^{2}=$0.3): (**a**) average time cost; (**b**) total energy cost.

Figure 6 reveals the performance of the algorithms for various energy budgets ρ. The figures indicate that by increasing the energy budget ρ, all algorithms will achieve similar performance as the time greedy algorithm. Users will have enough energy budget to neglect cost and stick to the BS with the lowest average time cost. It must be noted that if users do not have BS-side information they will experience performance loss (see Figure 6).

Therefore, the EMM-LSI and proposed BS-learning algorithms are not going to reach the optimum offline cost level. As a result, EMM-LSI and the proposed BS-learning algorithms will not reach the optimum offline cost level. The next important point is the slope of the lines, as shown in Figure 6. The results indicate that our proposed algorithms are near optimum for the offline solution compared to the algorithms in [43].

## 7. Conclusions

Significant research has been accomplished to determine online task offloading decisions in MEC while considering user mobility, and a limited energy budget has been allocated to users. The optimisation function has been developed as a trade-off between the time cost and energy cost, not to exceed the energy budget. The online algorithms are considered as potential solutions as future upcoming tasks are unknown. In this manuscript, two scenarios have been presented. The first scenario is when both the user-side and BS-side information are available. The user will be able to calculate the optimum BS and remain for the entire task processing period. The second scenario is when users have no access to BS information. Unlike the first scenario, users cannot calculate the utility cost and make offloading decisions based on that. Instead, users will offload limited tasks to each BS and observe the total time cost and energy consumption. After determining the optimum BS, the remaining tasks are offloaded to that BS. The BS may change during task offloading due to sub-optimum BS selection in the BS-learning algorithm, resulting in additional costs and HOs. The simulation results indicate that our two proposed algorithms perform slightly better than those in existing literature. Additionally, a performance loss is provided in the second scenario due to HO, and the outcomes are similar to the optimal offline solution.

In future work, it would be better to consider resource allocation in the problem formulation. This requires solving NP-hard and non-convex problems. One potential solution is to use machine learning (ML) approaches [63,64]. Applying ML approaches in MEC systems will be challenging because ML algorithms are extremely complex and computation resources are in high demand. Dividing learning computations into smaller tasks and distributing them among multiple MEC servers can be a possible solution. The issues would then be to determine the types of computations that can be divided as well as the method to divide while considering MEC resources. Merging the results obtained from different MEC servers for a specific task should also be managed and assessed. Due to UE mobility and frequent handovers among MEC servers, it is quite challenging to integrate outputs from various sub-tasks into a single output when the UE’s trajectory and location are dynamic. Future work should employ deep neural network (DNN) with multi-objective functions to optimise HO and computation offloading. Additionally, for the scenario where the UEs are far from network coverage, we can implement a device-to-device (D2D) connection or use MEC servers inside vehicular systems. Furthermore, for simplicity, multipath propagation was not considered here, and future work needs to consider the multipath impact into the problem formulation. Furthermore, the BSs in both algorithms are assumed to be all activated during the offloading process. Recent technologies such as software-defined networking (SDN) require a programmable network that enables developers to switch ON/OFF the BSs. In future work, it would be better to integrate the SDN with the MEC technology. Finally, future work could also analyse the run-time complexity of algorithms and compare it with benchmarks.

## Figures and Tables

**Table 3:** Employed simulation parameters with corresponding values.

Parameters	Value
Radius of the BS coverage area *R*	150 m
Availbe CPU frequency on BS $F_{n}$	25 GHz
Channel bandwidth ω	20 MHz
Channel gain from UE to BS $h_{m,n}$	127+30logd(km) dB
Subtask size	$d_{sub}=0.62$ Mbits
Input data size of each task $d_{m}$	[37.2,74.4] Mbits
Computation intensity of each task $c_{m}$	[500,1000] cycles/bit
Total available computation CPU for each task *m* by BS *n* $f_{m,n}$	$\left[0,F_{n}\right]$
Computation deadline of each task $t_{m}$	150 ms
Noise power $N_{\sigma^{2}}$	$2\times 10^{-13}$ W
UE transmission power $p_{m}$	0.5 W
One-time handover delay $\tau_{m}^{h}$	5 ms
Battery capacity	1 kJ

## Data Availability

Not applicable.

## Abbreviations

The following abbreviations are used in this manuscript:

3GPP	Third Generation Partnership Project
5G	Fifth Generation of Mobile Networks
6G	Sixth Generation of Mobile Networks
BS	Base Station
BSA	Boundless Simulation Area
DNN	Deep Neural Network
DRL	Deep Reinforcement Learning
ETSI	European Telecommunications Standards Institute
HO	Handover
IoT	Internet of Things
ML	Machine Learning
MDP	Markov Decision Process
MEC	Multi-access Edge Computing
RAN	Radio Access Network
RSU	Roadside Unit
RL	Reinforcement Learning
SDN	Software-defined Network
TO	Task Offloading
UDN	Ultra Dense Network
UE	User Equipment
VEC	Vehicular Edge Computing
